# Bio-guided Isolation of Antioxidant Compounds from *Chrysophyllum perpulchrum*, a Plant Used in the Ivory Coast Pharmacopeia

**DOI:** 10.3390/molecules15096386

**Published:** 2010-09-13

**Authors:** Bidie Alain Philippe, Ndjoko Karine, Attioua Koffi Barthélemy, Zirihi Guédé Noél, N’guessan Jean David, Djaman Allico Joseph, Kurt Hosttetmann

**Affiliations:** 1 Laboratoire de Pharmacodynamie-Biochimique; UFR Biosciences; Université de Cocody, 22 BP 582 Abidjan 22, Ivory Coast; 2 Laboratoire de Pharmacognosie et de Phytochimie de l’Université de Genève, Quai Ernest-Ansermet 30/CH-1211 Geneva 4, Switzerland; 3 Laboratoire de Chimie Organique Biologique; UFR SSMT; Université de Cocody, 22 BP 582 Abidjan 22, Ivory Coast; 4 Laboratoire de Botanique; UFR Biosciences; Université de Cocody, 22 BP 582 Abidjan 22, Ivory Coast

**Keywords:** *Chrysophyllum perpulchrum*, Sapotaceae, catechin, dimeric procyanidins, antioxidant activity, free radical scavenging activity, quercetin

## Abstract

*Chrysophyllum perpulchrum* (Sapotaceae) is used in the traditional Ivory Coast pharmacopeia to cure fevers. The extract of *C. perpulchrum* used for this study was the powdered form obtained from the maceration of the dried plant bark in 96% methanol, followed by evaporation to dryness. In the present study, the antioxidative and radical-scavenging activities of the methanolic extract were studied with three standard biological tests: DPPH reduction, ferric thiocyanate (FTC) lipidic peroxidation inhibition and thiobarbituric acid reacting substances (TBARS). Gallic acid and quercetin were used as references. The total amount of phenolic compounds in the extract was determined by ultraviolet (UV) spectrometry and calculated as gallic acid equivalents. Catechin and two dimeric procyanidins were found to be the compounds responsible for the activities. They were chemically dereplicated in the extract by LC-MS. For quantitation purposes, they were isolated by successive chromatographic methods and characterized by mass spectrometry (MS) and nuclear magnetic resonance (NMR) spectrometry. The quantities of these compounds in *C. perpulchrum* were 5.4% for catechin (**P1**), and 5.6 and 9.2% for dimers (compounds **2 (P2)** and **3 (P3**)), respectively. They displayed antioxidant activity with IC_50_ values of 2.50 ± 0.15 µg/mL (**P1**), 2.10 ± 0.2 µg/mL (**P2**) and 2.10 ± 0.1 µg/mL (**P3**). The total extract, the active fractions and the pure compounds inhibited the lipid peroxidation by the FTC method and the TBARS method in the range of 60%. These values were comparable to those seen for quercetin.

## 1. Introduction

There are numerous conditions under which oxidative stress can become a real heath problem. Defined as the imbalance in the balance of antioxidant/free radicals towards the latter, oxidative stress causes extensive damage to biological molecules such as DNA, lipids and proteins. Thus it is the cause of several diseases including cancer, cataracts, amyotrophic lateral sclerosis, accelerated aging, the acute respiratory distress syndrome and pulmonary oedema [1].

In view of the significant damage occasioned by oxidative stress, in recent years researchers have undertaken the search for antioxidant compounds to combat it. Currently a variety of synthetic antioxidant supplements are marketed to balance oxidants/antioxidants, but unfortunately these substances have suspected toxic effects. This is the case of butylated hydroxyanisole (BHA), butylated hydroxytoluene (BHT) and gallic acid esters, all of which are suspected of having negative heath effects [2] and consequently, strict restrictions on the use of these substances have been implemented in several countries such as Japan, Australia, Rumania, Sweden [3,4] and Pakistan. In Pakistan, for example, their use is prohibited for their endocrine disruptive effects [5]. In addition these synthetic antioxidants have shown low solubility and only moderate antioxidant activity [6]. For these reasons, in recent years researchers have focused on natural antioxidants, including those obtainable from plants. According to Cook and Samman [7], the antioxidant activities of plants are mainly due to the presence of secondary metabolites of the polyphenol class. These antioxidants can play an important role in the prevention and therapy of various diseases. In order to provide a solution to this problem, we propose to contribute to the search for new antioxidants. Thus, in this study, the chemical description and the biological properties of *C. perpulchrum*, a plant from the Ivory Coast pharmacopoeia was investigated in order to find a possible new indigenous natural source of antioxidants. *C. perpulchrum* is a tallish tree, often fluted at its bottom. The plant is found in Liberia, Cameroon as well as Uganda [8]. In Ivory Coast, it mainly inhabits forests from Amotioro (Tiassale region) Saioua (Issia department). It has many traditional usages. The fruit is edible and the juice is used locally to treat malaria. To our knowledge, the plant has not yet been studied extensively. 

## 2. Results and Discussion

### 2.1. Structural determination of the isolated compounds

Compound **1** (**P1)** was isolated as a yellow powder with a molecular formula C_15_H_14_O_6_ (*m/z* 290.0) established by LC-ESIMS. The infrared spectrum showed a hydroxyl group absorption band at 3.350 cm^−1^. The ^1^H- and ^13^C-NMR spectra showed evidence of a flavonoid, with signals at: 4.84 (H-2, 1H, *d*, *J* = 3.4 Hz), 4.78 (H-3, 1H, *m*), 2.834 (H-4, 1H, *m*); 2.527 (H-4, 1H, *m*), 5.85 (H-6, 1H, *d*, *J =* 3.4 Hz), 5.92 (H-8, 1H, *d*, *J =* 3.4 Hz) 6.82 (H-2’, 1H, *dd*, *J =* 2, 5.5 Hz), 6.75 ppm (H-3’; H-6’, 2H, *dd; J =* 2, 5.5 Hz) and at 84.9 (C-2), 70.9 (C-3), 159.0 (C-5), 159.6 (C-7), 159.6 (C-9) and 148.3 (C-4’ and C-5’) ppm. The UV spectra showed two absorption bands at 240 and 280 nm, which further confirmed that **P1** was a flavonoid. The full ^1^H- and ^13^C-NMR assignments were established by a combination of HSQC, COSY and HMBC correlations. From these observations and by comparison with literature NMR data [9], we were able to conclude that **P1** is catechin. 

Compound **2** (**P2)** was also isolated as a yellow powder with a molecular formula C_36_H_36_O_17_ (*m/z* 740.1) established by LC-ESIMS. The UV spectra showed three absorption bands at 226, 260 and 295 nm, which confirmed that **P2** belonged to the flavonoid glycoside series. It ^1^H- and ^13^C-NMR spectra were similar to those of **P1**, with a small difference due to the presence of a glycoside group: 4.78 ppm (H-3”, ^1^H, m), 5.03 ppm (H-6a, ^1^H, m) and 70.6 ppm (C-3”) and 106.5 ppm (C-6a). The position of the glycoside group was established as C-3” by the HSQC, HMBC and COSY correlations. The complete structure was established by comparison of **P2**’s ^1^H- and ^13^C-NMR data with those described in the literature [10,11]. In the ESI spectrum, peaks at *m/z* 289.0 and 578.9 correspond to the molecular weight (M-1) of catechin and its dimer 2(M-1), respectively. Another (weak) peak at *m/z* 738.9 corresponds to dimer + hexose. **P2** was thus identified as *2-(3,4-dihydroxyphenyl)-4-(2-(3,4-di-hydroxyphenyl)-5,7-dihydroxy-3-(3,4,5-trihydroxy-6-(hydroxymethyl)-tetrahydro-2H-pyran-2-yloxy)- 3,4-dihydro-2H-chromen-8-yl)-3,4-hydro-2H-chromene-3,5,7-triol.*

**Figure 1:**
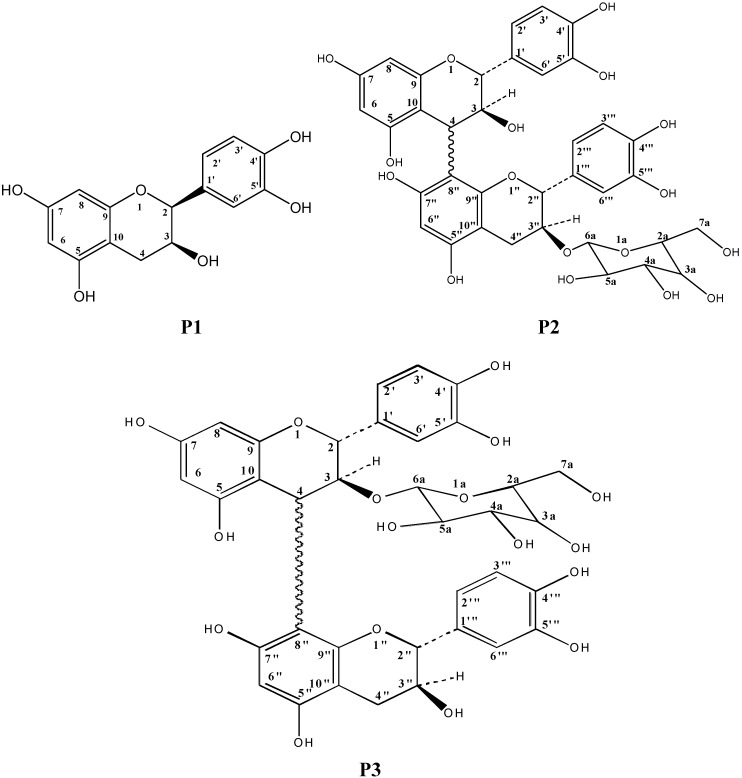
Chemical structure of compounds isolated from *Chrysophyllum perpulchrum.*

Compound **3** (**P3)** was identified as an isomer of **P2**, the difference being in the position of the glycoside on carbon C-3” for **P2** and C-3 for **P3**. Our spectral data for this compound were thus in complete conformity with those described in the literature [10,11] for *2-(3,4-dihydroxyphenyl)-8-(2-(3,4-dihydroxyphenyl)-5,7-dihydroxy-3-(3,4,5-trihydroxy-6-(hydroxymethyl)-tetrahydro-2H-pyran-2-yloxy)-3,4-dihydro-2H-chromen-8-yl)-3,4-hydro-2H-chromene-3,5,7-triol*. The structures of **P1-P2-P3** are shown in Figure 1. Complete ^1^H- and ^13^C-NMR assignments are given in Table 1.

### 2.2. Quantification and antioxidant activity

The different contents of catechin (**P1**) and catechin dimers 1 and 2 (**P2** and **P3**) from *C. perpulchrum* (Table 2) were calculated using an analytical HPLC calibration curve whose equation is:

y = 7074.8 x

and whose linearity was validated from 0 to 1 mg/mL. From this equation that we were able to determine from the peak areas the quantities of each purified compound (**P1**, **P2** and **P3**) present, which were 5.4%, 5.6% and 9.2% for **P1**, **P2** and **P3**, respectively (Table 2). The total content of catechin (20.2%) in the extract was significantly high in comparison to the amount found in some green teas [12,13]. 

Before the present study, there has been no work describing the antioxidant activity of *C. perpulchrum*. This activity was first tested using the 2,2’-diphenyl-1-picrylhydrazide (DPPH) model system. DPPH, a stable free radical, is commonly used as a reagent to evaluate the free radical scavenging activity of antioxidant substances. The antioxidant activity of the extract could be attributed to the presence of phenols (Table 3) which posses the ability to donate hydrogen radicals to DPPH for it to become a stable diamagnetic molecule. The concentration of compounds (substances) discoloring 50% of the DPPH values (IC_50_) for the extract and for the related compounds were calculated to be around 2.00 ± 0.25 µg/mL (Table 4). The values for catechin were in the range of the reference flavonoid quercetin. The other two methods used to assay lipid peroxidation inhibition showed a reduction of more than 60% by the extract. This result is in good correlation with the data already published regarding the activity of catechin on lipid peroxidation [14]. Quercetin was also tested as reference and its value was measured at 70% (Table 5).

### 2.3. Total phenolics content

Phenols are very important constituents because of their radical scavenging ability due to their hydroxyl groups [15]. The phenolics content may contribute directly to the antioxidant action [16]. It has been suggested that polyphenolic compounds have inhibitory effect on mutagenesis and carcinogenesis in humans [17]. Consequently, the antioxidant activities plant/herb extracts are often explained by their total phenolics and flavonoid contents with good correlation. The total phenolic content in the methanolic total extract of *C. perpulchrum* and its different fractions are given in Table 3.

### 2.4. DPPH activity of the methanolic extract of C. perpulchrum and of the fractions

Our results show a similar activity between quercetin, our reference molecule, and the total extract of *C. perpuchrum*, the different fractions (F4 and RP12) and purified compounds (**P1**, **P2** and **P3**). Indeed, the scavenging activity shown here by the DPPH test (Table 4) shows an IC_50_ decrease in total extract (IC_50_ = 4.00 ± 0.12 µg/mL) and purified compounds (IC_50_ = 2.50 ± 0.15 µg/mL; IC_50_ = 2.10 ± 0.09 µg/mL and IC_50_ = 2.05 ± 0.10 µg/mL). These values are roughly equal to those of quercetin (IC_50_ = 2.00 ± 025 µg/mL). 

### 2.5. Inhibition of lipid peroxidation by the FTC method and TBARS

We evaluated the inhibition of lipid peroxidation by the extract, fractions, the purified products and quercetin (reference). Our results (Table 5) indicate that the total extract, F4, RP12 and the purified products have percentages of inhibition similar to that of quercetin (70.02 **±** 3.89%). Similarly, we also assessed inhibition of lipid peroxidation by the TBARS method. The results (Table 5) show that for the lipid peroxidation with FTC, the total extract, F4, RP12, **P1**, **P2** and **P3** have percentages of inhibition very similar to that of quercetin (70.02 ± 3.89%) according to the statistical analysis. Concerning, the lipid peroxidation with TBARS, only, P3 has the similar percentage of inhibition to that of quercetin (81.55 ± 2.22%) according to the statistical analysis.

### 2.6. Correlations between total phenolic content and antioxidative function

Besides the DPPH [12], the estimation of the antioxidant activities has been also done by a lipidic peroxidation assay with FTC and TBARS [13]. The antioxidant activity of total extract, fractions and isolated and purified compounds from *C. perpulchrum* obtained after isolation followed by bio-guided purification was assessed by the percentage reduction of DPPH, total phenolic content, the percentage of inhibition of lipidic peroxidation obtained from the test on FTC and TBARS. The total methanolic extract of *C. perpulchrum* contains flavonoids that reduce the DPPH radical (IC_50_ = 4.00 ± 0.12 µg/mL). These results agree with those of Duh *et al.* [16] and Lin *et al.* [13], respectively, who showed that roots and flowers of *Thonningia sanguinea* reduce DPPH. 

The excessive production of free radicals can cause direct damage to biological molecules [17]. The antioxidants substances of *C. perpulchrum* could fight against the formation of free radicals in diseases. According to Chen and Ho [18], phenolic compounds are widely distributed in tissues of plants where we find many free radicals and antioxidant molecules. Moreover, the fight against free radicals requires the intervention of radical scavengers. Some groups of plant secondary metabolites such as phenolic acids and flavonoids [19,20,21] have high antioxidant activities. Their mechanisms of action are trapping and chelating of free radicals [22]. Indeed, these metabolites have the peculiarity to give up easily an electron or a proton in order to neutralize free radicals [15]. Among these compounds, flavonoids have enormous antiradical and antioxidant potentials and their effects on nutrition and human health are considerable. The phytochemical screening showed that *C. perpulchrum* contains these two groups of compounds and their presence would justify the observed antioxidant activity. Moreover, our results reveal the presence of phenol groups in the total methanolic extract of *C. perpulchrum*, in fractions F4 and RP12 and isolated and purified compounds **1**, **2**, and **3**, which C. ould be the basis of their scavenging activity. Similar results were reported by other authors who have shown that there is a relationship between phenolic content and antiradical activity of plant extracts [13]. Also, the relationship between antiradical activity and reduction of DPPH radical, iron reduction and phenolic composition of the methanolic extract of leaves of *Syzgium cumini* has been shown by Rekka and Kourounakis [23].

As for lipid peroxidation, it is defined as the oxidative deterioration of polyunsaturated lipids [24]. Total methanolic extract, fractions F4, RP12 and isolated and purified compounds **1**, **2**, and **3** from *C. perpulchrum*all show a high antioxidative activity which results in an inhibition of lipid peroxidation. With the FTC method, all samples have an antioxidant activity of more than 60%. These samples have antioxidant activities similar to those of quercetin (70.02 ± 1.02%), a reference molecule. 

In the TBARS method, six of the fourteen samples (total extract, fractions and purified compounds) have an antioxidant activity of more than 60%. The results of lipid peroxidation by the TBARS method indicate no significant (ns) difference between the antioxidant activities of the six samples mentioned above. This suggests that in addition to phenolics and flavonoids, other classes of secondary metabolites may be implicated in the inhibition of lipid peroxidation. Non-phenolic compounds may also contribute by synergistic or antagonistic effects to the measured antioxidant activity of extracts. These results are consistent with those found by Hsu *et al.* [25]. 

In view of the foregoing, we can say that the four tests used to demonstrate the antioxidant and scavenging activities and all were significant enough and all move in the same direction. This suggests that *C. perpulchrum* might solve the problem of the presence of lots of free radicals in tissues. We have shown through this study that the three purified compounds (catechin and two dimers of catechin with glucose) from the total methanolic extract of *C. perpulchrum* have antioxidant and scavenging activities similar to that of quercetin, our reference molecule. 

## 3. Experimental

### 3.1. Chemicals and plant materials

#### 3.1.1. Plant material

Barks of *C. perpulchrum* were collected in the central-west forest region of Issia (Côte d’Ivoire) in December 2008. The plant was identified by Prof. Ake-Assi of the botanical gardens of the University of Cocody-Abidjan, where a numbered specimen (voucher N° 8375) was deposited. 

#### 3.1.2. Chemicals

Ascorbic acid (Vit.C), Tris-HCl, linoleic acid, iron sulfate, thiobarbituric acid (TBA), ferric thiocyanate (FTC), 2.2’-diphenyl-1-picrylhydrazyl (DPPH) and quercetin were purchased from Sigma-Aldrich (St. Louis, USA). Milli-Q water and HPLC grade CH_3_OH for analytical RP-HPLC, and other chemicals were of analytical reagent (AR) purity grade.

### 3.2. Extraction procedure

Plants were air dried at room temperature for 3 weeks to a constant weight. The dried plants were later ground to powder. Then, the dried material was grounded, pulverized and extracted with methanol. 50 g were mixed with 1.5 L of methanol 96% and agitated using a magnetic agitator (IKAMAG RCT) (Staufen, Germany) during 48 hours at room temperature. After cotton and paper (Watmann 3 mm filters) filtration, evaporation of the solvent was achieved with a rotatory evaporator (Büchi 461 w/water bath, Strasbourg, France) at 40 ºC, 10 g were obtained after evaporation (yield 20%).

### 3.3. Determination of total phenolic compounds

The total amount of phenolic compounds in the extract was determined and expressed as gallic acid equivalents per gram of extract. The Folin-Ciocalteu method [26] was used as followed: a volume of 0.5 mL of plant extract (concentration of 0.1 g/mL) were mixed with Folin-Ciocalteu phenol reagent (5 mL) diluted ten times with distilled water and sodium carbonate solution (4 mL, 1M). After 15 min of incubation at room temperature (25 ºC) the absorbance was measured using a Spectronic^®^ GENESYS 5 UV spectrometer (Spectronic Instruments, USA) at 765 nm. The calibration curve was obtained with a gallic acid standard solution prepared under the same conditions as the extract. The range of concentration was between 50–250 mg/mL. 

### 3.4. Free radical scavenging ability on 2.2-diphenyl-1-picrylhydrazyl

To assess the scavenging ability on 2.2-diphenyl-1-picrylhydrazyl (DPPH), each extract (0.1 mL, 15–250 g/mL) in methanol was mixed with methanol solution (3 mL) containing DPPH radicals (0.004%, w/w). Three replicates were carried out. The mixture was shaken vigorously and left to stand for 30 min in the dark before measuring the absorbance at 517 nm against a blank [27,28,29]. Then the scavenging ability was calculated using the following equation:

Scavenging ability (%) = [(A_517_ of control – A_517_ of sample) / A_517_ of control] × 100.

### 3.5. Measurement of lipid peroxidation

#### 3.5.1. Determination of hydroperoxides by the ferric thiocyanate (FTC) method 

The antioxidant activity against lipid peroxidation of plant extracts is measured by the inhibition of peroxidation of linoleic acid using the ferric thiocyanate method, as described in [29,30,31]. The reaction mixture containing respectively extracts (0.4 mL, 100 g/mL), linoleic acid (0.4 mL, 2.52% in absolute ethanol) and phosphate buffer (0.8 mL, pH 7.4) was incubated in a water bath for 1 hour at 40 ºC. An aliquot (0.1 mL) of this solution is then added to the mixture of 70% ethanol (5 mL) and ammonium thiocyanate (0.1 mL, 30%). After 3 min, a volume of FeCl_2_ (0.1 mL) prepared in 3.5% HCl (20 mM) was added to the reaction medium. A negative control is achieved by replacing the extracted by distilled water. The absorbance of the red color of the resulting solution is read for 7 days to 500 nm in a spectrophotometer every 24 hours until the absorbance negative control (distilled water) reaches its maximum. The percentage of inhibition of lipid peroxidation is then calculated using the following equation:

Inhibition (%) = (1 − (OD sample / OD negative control)) × 100

#### 3.5.2. Determination of Thiobarbituric Acid-reacting Substances (TBARS) 

The method of Choi *et al.* [33] using an induction of lipid peroxidation by ascorbic acid/iron sulfate (Fenton reaction) has been adapted for this test. To a sample of plant extract (600 μL, 500 g/mL) was added Tris-HCl buffer (300 μL, pH 7.5, 20 mM), 20 mM linoleic acid (500 μL) and ferric sulphate (100 μL, 4 mM). Peroxidation begins after addition of 5 mM ascorbic acid (100 μL). Each reaction mixture obtained is incubated in a water bath at 37 ºC for 60 min. After this step, TCA (2 mL, 10%) are added to all tubes. Then, to an aliquot (1 mL) collected in each of reaction mixtures prepared previously was added TBA (1 mL, 1%). The new reaction mixtures obtained were placed in a boiling water bath at 95 ºC for 20 min. Gallic acid and distilled water respectively were used as reference molecule and negative control. The absorbance was read in a spectrophotometer at 532 nm and percentage inhibition of linoleic acid is determined by the following equation:

Inhibition (%) = (1 − (OD sample / OD negative control)) × 100

### 3.6. LC-MS analysis

Liquid chromatography was performed with a HPLC system equipped with a binary pump and photodiode array high speed spectrophotometric detector and autosampler, all controlled by the Agilent Chemstation software (Agilent, Palo Alto, CA, USA). A Waters Symmetry C_18_ (15 mm × 4.6 mm i.d.; 5 µm) column was used for the separation with MeCN-H_2_O (0.1% formic acid) as mobile phase; the separation employed a gradient that began with 5:95 (MeCN-H_2_O) and ended with 95:5 (MeCN-H_2_O). The total run time was 30 min. The column was washed with 100 % (MeCN) during 5 min. The flow rate was maintained at 1 mL/min. ESI-MS detection was achieved in negative mode on a Finnigan (San Jose, CA, USA) model LCQ ion trap spectrometer equipped with a Finnigan ESI source. The ESI parameters were as follows: spray voltage, 3.5 kV; capillary transfer temperature, 200 ºC; nebulisation gas (nitrogen), 70 psi. In source collision-induced dissociation of 15 eV was used. For MS/MS experiements, the collision energy was fixed at 40%. 

### 3.7. Isolation and characterization of catechin and its derivatives

The extract was submitted to a gel filtration on polyamide phase with a mixture of EtOH-H_2_O in different portions: 30/70, 50/50, 70/30 and 96/04 (V/V). A portion (100 mg) of the active fraction F4 were further chhromatographed by vacuum liquid chromatography on a RP_18_ column eluted with MeOH-H_2_O under the following gradient conditions: 5/95; 15/85; 35/65; 55/45; 80/20; 100/0 (v/v). Catechin and it derivatives were isolated from the two first fractions using semi-preparative HPLC carried out with a LC-8 pump equipped with a SPD-10AVP detector (Shimadzu) using a XTerra prep-MS C_18_ ODB column (5 µm, 19 × 150 mm; Waters), and a MeOH + 0.1% formic acid (FA)/H_2_O + 0.1% FA gradient (20:80–40:60–100:0; 10 mL/min, in 28 min). Detection was done at 275 nm. Their characterization was done by NMR spectroscopy and the data were compared with literature. The NMR spectra were acquired on a 500 MHz Unity Inova spectrometer (499.87 and 125.70 MHz for respectively ^1^H and ^13^C) working under the VNMR 6.1C software (Varian).

### 3.8. Statistical analysis

Experiments were run in duplicate, and statistical analyses were performed using the Graph Pad Prism 5.0 software. Analyses of variance (ANOVA) were conducted and multiple comparisons between means were performed by Tukey and Dunnett procedure. Significance level was defined as P < 0.05. Results were given as means **±** standard deviation.

## 4. Conclusions 

At the end of this study, we consider that the bio-guided purification of *C. perpulchrum* revealed three compounds with excellent proven antioxidant and scavenging activities. Thus, the study went from total extract of *C. perpulchrum*, to purified compounds through the various fractions showingand confirming at each stage the presence of antioxidant and scavenging activities. Furthermore, we found that compounds 1 (catechin), 2 and 3 (dimers of catechin + hexose) have antioxidant and scavenging activities similar to those of controls used (gallic acid and quercetin) and the compounds obtained are present in relatively large proportions in *C. perpulchrum* (**P1**: 5.4%; **P2**: 5.6% and **P3**: 9.2%). 

## Figures and Tables

**Table 1:** ^13^C- (125 MHz) and ^1^H-NMR (500 MHz) spectroscopic data for **P1**, **P2** and **P3.** Chemical shifts are given in ppm; multiplicities and coupling constants *J* (in parentheses) in Hz.

Position	P1			P2			P3	
	C	H		C	H		C	H
1	-	-		-	-		-	-
2	84.9	4.84(d, 3.4)		84.4	4.84(d,3.4)		80.5	5.29, (d, 3.4)
3	70.9	4.78(m)		70.6	4.78(m)		68.7	4.50 (m, )
4	30.5	4α: 2.834(m) 4β: 2.527(m)		29.9	4.13(d, 4.3)		26.2	4.37 (d, 4.2)
5	159.0	-		159.1	-		155.8	-
6	98.4	5.85(s)		97.9	5.85(s)		96.2	5.71 (s)
7	159.9			158.8	-		154.7	-
8	97.5	5.92(s)		96.8	-		105.1	5.75 (s)
9	159.6	-		159.2	-		155.3	-
10	102.9	-		102.2	-		101.8	-
1’	134.3	-		134.1	-		132.5	-
2’	122.1	6.82(d, 8.5)		122.8	6.82(d, 8.5)		122.2	6.80 (d, 8.5)
3’	118.1	6.75(d ; 8.5 )		118.0	6.75(d, 8.5 )		117.5	6.50(d, 8.5)
4’	148.3	-		148.1	-		144.6	-
5’	148.3	-		148.1	-		147.2	-
6’	117.3	6.75(d; 8.5)		118.0	6.75(d; 8.5)		115.2	6.50 (d, 8.5)
1’’	-	-		-	-		-	-
2’’				80.5	5.29, (d, 3.4)		84.4	4.84(d, 3.4)
3’’				68.7	4.12 (m)		70.6	4.78(m)
4’’				26.2	4’’α: 2.81 (m) 4’’β: 2.56 (m)		29.9	4α: 2.83(m) 4β: 2.57(m)
5’’				155.8	-		159.1	-
6’’				96.2	5.71 (s)		97.9	5.85(s)
7’’				154.7	-		156.8	-
8’’				105.1	-		105.2	-
9’’				155.3	-		159.2	-
10’’				101.8	-		102.2	-
1’’’				132.5	-		134.1	-
2’’’				122.2	6.80 (d, 8.5)		122.8	6.82(d, 8.5)
3’’’				117.5	6.50(d, 8.5)		118.0	6.75(d, 8.5 )
4’’’				144.6	-		148.1	-
5’’’				147.2	-		148.1	-
6’’’				115.2	6.50 (d, 8.5)		118.0	6.75(d; 8.5)
1a				-				
2a				81.2	3.76 (m)		81.2	3.76 (m)
3a				73.2	3.40(m)		73.2	3.40(m)
4a				77.5	3.50 (m)		77.5	3.50 (m)
5a				75.6	3.73 (m)		75.6	3.73 (m)
6a				106.5	5.03 (d, 6.5)		106.5	5.03 (d, 6.5)
7a				65.3	3.54 (m)		65.3	3.54 (m)

**Table 2:** Different amounts of purified compounds in *C. perpulchrum*.

Peaks	Peak areas	Concentration (mg/mL)	Amount (%)
Catechin (P1)	402.5	0.054	5.4
Dimer 1 (P2)	417.7	0.056	5.6
Dimer 2 (P3)	653.7	0.092	9.2

**Table 3:** Total phenolic content of *C. perpuchrum* and of fractions.

Samples	Total phenolic content (mg GAE/g)
**Total Extract**	**74.00 ± 0.68 a**
F1	0.42 ± 0.02 c
F2	1.48 ± 0.03 c d
F3	7.03 ± 0.15 e
**F4**	**77.57 ± 0.56 ab**
**RP12**	**80.62 ± 0.57 b**
RP3	31.76 ± 1.67
RP4	21.91 ± 0.78
RP5	8.62 ± 0.88 e
RP6	5.05 ± 0.40 d e

Letters a, b, c, d, e indicate significant differences; the same letters mean not significant variance in the value; We used Tukey’s test; F1; F2; F3 and F4 refer to fractions 1, 2, 3 and 4 obtained after chromatography on polyamide; RP12: refers to fractions 1 and 2 obtained after chromatography on a RP_18_ reverse phase column; RP3, RP4, RP5 and RP6 refer to fractions 3, 4, 5 and 6 obtained after chromatography on a RP_18_ reverse phase column; mg GAE/g: milligrams of equivalent of gallic acid per gram of dry matter.

**Table 4:** DPPH activity of the methanolic extract of *C. perpulchrum* and of the fractions.

Samples	Quercetin	Extract	F4	RP12	P 1	P 2	P 3
IC_50_ (µg/mL)	2.00 ± 0.25	4.00 ± 0.12	3.92 ± 0.05	3.5 ± 0.19	2.5 ± 0.15 ns	2.10 ± 0.09 ns	2.05 ± 0.10 ns

ns: not significant; We used Dunnett’s test; µg/mL: microgram per millilitter; IC_50_: Concentration of compounds (substances) discoloring 50% of the DPPH.

**Table 5:** Lipidic peroxidation with FTC and TBARS.

Samples	Inhibition (%) / FTC	Inhibition (%) / TBARS
**Quercetin**	**70.02 ± 3.89**	**81.55 ± 2.22**
**Total Extract**	**64.57 ± 1.96**	**64.40 ± 1.22**
**F1**	54.37 ± 222	54.35 ± 0.89
**F2**	52.03 ± 0.89	51.10 ± 1.96
**F3**	51.87 ± 0.89	54.00 ± 2.22
**F4**	**67.03 ± 1.22 ns**	**70.30 ± 3.89 ns**
**RP12**	**68.02 ± 0.78 ns**	**75.75 ± 0.85 ns**
**P1**	**67.12 ± 2.55 ns**	**77.6 ± 389 ns**
**P2**	**68.02 ± 2.89 ns**	**78.25 ± 2.56 ns**
**P3**	**68.85 ± 1.96 ns**	**80.10 ± 3.89 ns**

ns: not significant; We used Dunnett’s test; P1, P2, P3 refer to purified compounds from *C. perpulchrum*.; P1: catechin; P2: Dimer 1; P3: Dimer 2.

## References

[1] Mates J.M., Sanchez-Jimenez F.M. (2000). Role of reactive oxygen species in apoptosis: implications for cancer therapy. Int. J. Biochem. Cell Biol..

[2] Davia M.L., Gnudil F. (1999). Phenolic compounds in surface water. Wat. Res..

[3] Blasucci L. All Health’s Breaking Loose: FYI on BHT..

[4] Answers.com Dictionary Butylated hydroxytoluene..

[5] Ejaz S., Akram W., Lim C.W., Lee J.J., Hussain I. (2004). Endocrine discrupting pesticides: a leading cause of cancer among rural people in Pakistan. Exp. Oncol..

[6] Barlow S.M., Hudson B.J.F. (1990). Toxicological aspects of antioxidants used as food additives. Food Antioxidants.

[7] Cook N.C., Samman S. (1996). Flavonoids-chemistry, metabolism, cardiodepressive effects, and dietary sources. Nutr. Biochem..

[8] Louppe D., Oteng-Amoako A.A., Brink M. (2008). Ressources Végétales de l’Afrique Tropicale 7(1): Bois d’oeuvre 1.

[9] Escribano-Bailón T., Olivier D., Brouillard R. (1996). Coupling reactions between flavylium ions and catechin. Phytochemistry.

[10] John L., Phyllis, Coley D., Thomas, Kursar A. (2004). Cinnamoyl glucosides of catechin and dimeric procyanidins from young leaves of *Inga umbellifera* (Fabaceae). Phytochemistry.

[11] Torgils F., Saleh R., Øyvind M.A. (2004). Dimeric anthocyanins from strawberry (*Fragaria ananassa*) consisting of pelargonidin 3-glucoside covalently linked to four flavan-3-ols. Phytochemistry.

[12] Cabrera C., Artacho R., Gimenez R. (2006). Beneficial effects of green tea - a review. J. Am. Coll. Nutr..

[13] Lin Y.S., Tsai Y.J., Tsay J.S., Lin J.K. (2003). Factors affecting the levels of tea polyphenols and caffeine in tea leaves. J. Agric. Food Chem..

[14] Neiva T.J.C., Morais L., Polack M., Simoes C.M.O., D'amico E.A. (1999). Effects of catechins on human blood platelet aggregation and lipid peroxidation. Phytother. Res..

[15] Hatano T., Edamatsu R., Hiramatsu M., Mori A., Fujita Y., Yasuhara T., Yoshida T., Okuda T. (1989). Effect of inreaction of tannins with co-existing substances VI. Effect of tannins and related polyphénols on superoxide anion radical and on DPPH radical. Chem. Pharm. Bull..

[16] Duh P.D., Tu Y.Y., Yen G.C. (1999). Antioxidant Activity of Water Extract of Harng Jyur (*Chrysanthemum morifolium* Ramat). Leb-ensm. Technol..

[17] Tanaka M., Kuei C.W., Nagashima Y., Taguchi T. (1998). Application of antioxidative maillard reaction products from histidine and glucose to sardine products. Nippon Sui. Gakk..

[18] Chen C.W., Ho C.T. (1995). Antioxidant properties of polyphenols extracted from green tea and black tea. J. Lipids.

[19] Gyamfi M.A., Aniya Y. (1998). Medicinal herb, *Thonningia sanguinea* protects against aflatoxin B-1 acute hepatotoxicity in Fischer 344 rats. Hum. Expl. Toxicol..

[20] N’guessan J.D., Zirihi G.N., Kra A.K.M., Kouakou K., Djaman A.J., Guedé-Guina F. (2007). Free radical scavenging activity, flavonoid and phenolic contents of selected Ivoirian plants. Int. J. Nat. Appl. Sci..

[21] Mc Donald S., Prenzler P.D., Autolovich M., Robards K. (2001). Phenolic content and antioxidant activity of olive extracts,. Food Chem..

[22] Favier A. (2003). Le stress oxydant: Intérêt conceptuel et expérimental dans la compréhension des mécanismes des maladies et potentiel thérapeutique. L’actualité Chim..

[23] Rekka E., Kourounakis P.N. (1991). Effect of hydroxyethyl rutosides and related compounds on lipid peroxidation and free radical scavenging activity. Some structural aspects. J. Pharm. Pharmacol..

[24] Sergeant C., Hamon C., Simonoff M., Constans J., Conri C., Peuchant C., Delmas- Beauvieux M.-C., Clerc C., Pellegrin J.L., Leng B., Pellegrin I., Fleury H., Montagnier L., Olivier R., Pasquier C. (1998). Oxidative Stress in Cancer, AIDS and Neurodegenerative Diseases.

[25] Hsu C.Y., Chan Y.P., Chang J. (2007). Antioxidant activity of extract from *Polygonum cuspidatum*. Biol. Res..

[26] Caï Y.Z., Mei S., Jie X., Luo Q., Corke H. (2006). Structure-radical scavenging activity relationships of phenolic compounds from traditional Chinese medicinal plants. Life Sci..

[27] Kessler M., Ubeaud G., Jung L. (2003). Anti- and pro-oxidant activity of rutin and quercetin derivatives. J. Pharm. Pharmacol..

[28] Zhi P.R., Liang L.Z., Yi M.L. (2008). Evaluation of antioxydant activity of Syzygium cumini leaves. Molecules.

[29] Takao T., Kitatani F., Watanabe N., Yagi A., Sakata K.A. (1994). Simple screening method for antioxidant and isolation of several antioxidant produced by marin bacteria from fish and shellfish. Biosci. Biotechnol. Biochem..

[30] Kappus H., Aruoma O.I., Halliwell B. (1991). Lipid peroxidation-Mechanism and biological relevance. Free radicals and Food Additives.

[31] Kahkonen M.P., Hopia A.L., Viorela H.J., Ranha J.P., Pihlaja K., Kujula T.S. (1999). Antioxidant activities of plants extracts containing phenolic compounds. J. Agric. Food Chem..

[32] Choi C.W., Kim S.C., Hwang S.S., Choi B.K., Ahn H.J., Lee M.Z., Park S.H., Kim S.K. (2002). Antioxidant activity and free radical scavenging capacity between Korean medicinal plant and flavonoids by assay-guided comparison. Plant Sci..

[33] Garcia A.M., De Pascual-Teresa S., Santos-Buelga C., Rivas-Gonzalo J.C. (2004). Evaluation of antioxydant properties of fruits. Food Chem..

